# Translating potential improvement in the precision and accuracy of lung nodule measurements on computed tomography scans by software derived from artificial intelligence into impact on clinical practice—a simulation study

**DOI:** 10.1093/bjrai/ubae010

**Published:** 2024-06-06

**Authors:** Mubarak Patel, Peter Auguste, Jason Madan, Hesam Ghiasvand, Julia Geppert, Asra Asgharzadeh, Emma Helm, Yen-Fu Chen, Daniel Gallacher

**Affiliations:** Applied Health, Warwick Medical School, University of Warwick, Coventry, CV4 7AL, United Kingdom; Applied Health, Warwick Medical School, University of Warwick, Coventry, CV4 7AL, United Kingdom; Applied Health, Warwick Medical School, University of Warwick, Coventry, CV4 7AL, United Kingdom; Research Centre for Healthcare & Communities, Research Institute for Health and Wellbeing, Coventry University, Coventry, CV1 5FB, United Kingdom; Applied Health, Warwick Medical School, University of Warwick, Coventry, CV4 7AL, United Kingdom; Bristol Medical School, University of Bristol, Bristol, BS8 2BN, United Kingdom; Radiology, University Hospitals Coventry and Warwickshire, Coventry, CV2 2DX, United Kingdom; Applied Health, Warwick Medical School, University of Warwick, Coventry, CV4 7AL, United Kingdom; Applied Health, Warwick Medical School, University of Warwick, Coventry, CV4 7AL, United Kingdom

**Keywords:** artificial intelligence, simulation, measurement accuracy, lung nodule, chest CT, precision, nodule growth

## Abstract

**Objectives:**

Accurate measurement of lung nodules is pivotal to lung cancer detection and management. Nodule size forms the main basis of risk categorization in existing guidelines. However, measurements can be highly variable between manual readers. This article explores the impact of potentially improved nodule size measurement assisted by generic artificial intelligence (AI)-derived software on clinical management compared with manual measurement.

**Methods:**

The simulation study created a baseline cohort of people with lung nodules, guided by nodule size distributions reported in the literature. Precision and accuracy were simulated to emulate measurement of nodule size by radiologists with and without the assistance of AI-derived software and by the software alone. Nodule growth was modelled over a 4-year time frame, allowing evaluation of management strategies based on existing clinical guidelines.

**Results:**

Measurement assisted by AI-derived software increased cancer detection compared to an unassisted radiologist for a combined solid and sub-solid nodule population (62.5% vs 61.4%). AI-assisted measurement also correctly identified more benign nodules (95.8% vs 95.4%); however, it was associated with over an additional month of surveillance on average (5.12 vs 3.95 months). On average, with AI assistance people with cancer are diagnosed faster, and people without cancer are monitored longer.

**Conclusions:**

In this simulation, the potential benefits of improved accuracy and precision associated with AI-based diameter measurement is associated with additional monitoring of non-cancerous nodules. AI may offer additional benefits not captured in this simulation, and it is important to generate data supporting these, and adjust guidelines as necessary.

**Advances in knowledge:**

This article shows the effects of greater measurement accuracy associated with AI assistance compared with unassisted measurement.

## Introduction

Lung nodules are rounded opacities, well or poorly defined, measuring up to 3 cm in diameter.^1^ They are common findings in chest computed tomography (CT) scans. Approximately 95% of pulmonary nodules identified are benign,^2^ but the remaining are cancerous and require action. There is a strong correlation between both the size and the growth of lung nodules and their risk of being malignant, and the clinical management of lung nodules is largely determined by their size and rate of growth. Accurate measurement of the size and growth of lung nodules is, therefore, a crucial element of the current diagnostic and management pathways relating to lung nodules and lung cancer.

The Lung CT Screening Reporting and Data System (Lung-RADS) was established by the American College of Radiology to standardize the interpretation, reporting, and management recommendations for low-dose lung cancer screening CT images.^3^ Used in countries such as the United States and other parts of the world, Lung-RADS categorizes nodules based on their type, appearance, size/volume and growth, and makes recommendations for appropriate action.

In the United Kingdom, the pathways for the detection and management of lung nodules as specified in the British Thoracic Society (BTS) guidelines^4^ are determined similarly by nodule features and nodule growth. Lung nodules are broadly classified as solid or sub-solid, depending on whether parts of the nodules have a different level of density on the CT scan.^4^ Solid nodules are more common than subsolid nodules; the latter carry a higher risk of cancer but are typically more indolent. Nodules with certain features such as calcification are unlikely to be malignant and are usually disregarded. In addition to nodule types and features, the size and growth of nodules are key determinants of the risk stratification which dictates subsequent clinical management.

Lung nodules are detected in different ways. Targeted screening of people deemed at higher risk (eg, based on smoking history, age, and family history of lung cancer) aims to detect cancerous nodules when they are asymptomatic and at an early stage. Nodules may also be detected following symptomatic presentation or following medical imaging performed for unrelated reasons.

Traditionally, nodule size is measured manually using an electronic callipers; for example, one-dimensional (1D) as the maximum nodule diameter, or two-dimensional (2D) measuring the longest and perpendicular diameters in the transverse plane.^5^ Accuracy and precision of these 1D and 2D methods are compromised by the underlying assumptions that nodules are spherical in shape and grow symmetrically.^6^ Measurement variability of electronic calliper-based diameter measurements is also caused by the fact that readers are typically required to select an axial section for which the nodule is estimated to be at its largest and then manually place cursors at the boundaries of the nodule. Intra-reader and inter-reader agreement in nodules less than 2 cm is poor.^7^ Size changes <1.32 mm for the same reader and <1.73 mm between readers cannot be distinguished from measurement errors, and nodule growth can confidently be determined only beyond these limits, which can result in misclassification of lung nodules and lead to suboptimal clinical management. More recently, 3D segmentation software has been used to perform volume measurement of lung nodules using semi-automated or automated techniques; these methods can better encapsulate the 3D nature of lung nodules, require less human interaction and, therefore, provide greater consistency in size measurement and resulting follow-up recommendations.^8–10^ In particular, software derived from artificial intelligence (AI) has shown promise in improving the precision and accuracy of nodule measurement^11^ and subsequent risk categorization.^12^^,^^13^

Advancements in AI have the potential to standardize lung nodule size measurement, increase accuracy/detection and streamline clinical decision-making. Computer-aided diagnostic (CAD) techniques can be used as a first reader, second reader or concurrently with a radiologist. It can also allow radiologists to consider the volume of lung nodules in three dimensions rather than the 2D measurements of nodule diameter, allowing measurements to be reproducible and less susceptible to intra- and inter-observer variability.^14^ A recent systematic review on AI assistance for lung nodule assessment in CT scans found that radiologists, with AI support, showed improved sensitivity and AUC in nodule detection, albeit with slightly reduced specificity. Additionally, AI assistance generally enhanced radiologists' sensitivity, specificity, and AUC in malignancy prediction.^15^ Further research also noted AI assistance significantly enhanced the detection rates of various sizes and types of lung nodules.^16^ Particularly noteworthy was the substantial increase in detecting nodules smaller than 5 mm, a challenge traditionally encountered in AI prediction.^17^ When comparing nodule sizes, CAD agreed with the reference standard (a consensus by three radiologists) in 67% of cases. However, it often overestimated measurements, sometimes due to errors like measuring adjacent arteries or selecting the wrong section.^17^

While improvement in precision (ie, reduced variability) associated with AI-assisted image analysis has been documented,^18^ how such improvement translates into changes in clinical management of patients is unclear. In addition, it has been shown that nodule measurement by AI-derived software tends to over-estimate nodule sizes compared with unassisted reading.^19^ This may affect the number of patients requiring CT surveillance, which could have implications on healthcare costs and patient anxiety. The overall impact of this potential change in measurement accuracy, in combination with the change in precision, therefore, requires further exploration. Our simulation study aims to address these questions through a simulation comparing manual measurement to AI-assisted measurement.

## Methods

We constructed hypothetical cohorts of nodules, with each nodule representing the risk-dominant nodule for one person (for someone who has more than one lung nodule, the risk dominant nodule is the one [usually the largest] which is considered to have the highest risk of malignancy and on which clinical management decisions are based) using available data on the distribution of sizes and types of lung nodules from the literature. We then simulated nodule growth and detection for a screening population.

### Nodule type

Lung nodules can be broadly classified into solid nodules or subsolid nodules depending on the level of density and other features in the CT image.^4^ We separately simulated 1 000 000 solid and 1 000 000 subsolid nodules, and combined them into a single sample of 1 000 000 nodules containing 93.9% solid nodules (based on a large study evaluating a screening programme in Korea.^20^). This article focuses on the combined population.

### Base case simulations

A systematic review^21^ (reported separately) identified papers to provide the majority of model inputs. The review was undertaken to inform a diagnostic technology appraisal by the UK National Institute for Health and Care Excellence and focused on AI-derived software which received regulatory approval (CE mark) by December 2021 or was anticipated to be commercially available in the United Kingdom by 2023. As no single AI software provided sufficient evidence required for our simulation, the model inputs were collated from best available data related to different AI software. As a result, our simulation represents a generic scenario of application of AI-assistance rather than results for a specific AI software.

No meta-analysis was performed in the systematic review as the included studies and data were too heterogeneous. Data required for the simulation were available from only one study for many of the parameters. Where data were potentially available from more than one study, they were selected on the basis of their applicability to the UK screening population, taking into account the sample sizes of the studies and risk of bias.

The parameters of the log-normal models used to sample the true initial nodule sizes were calculated by rearranging the formula for the quantiles of the log-normal distribution [eqn (1)] and then calculating SD based on the median and IQR, with nodules not exceeding 30 mm.

Equation 1: Formula used to calculate the standard deviation used in the simulation from the log-normal distribution

$$\mathrm{quantile}=\exp\left(\mu +\;\sqrt{2\sigma^{2}}{\mathrm{erf}}^{-1}\left(2x-1\right)\right);\mathrm{for}\;x\;\mathrm{in}\;\log\left(X\right)∼N(\mu ,\sigma )$$

where μ is the mean, ${\mathrm{erf}}^{-1}$ is the reciprocal of the error function, and σ is what is being calculated.

From the true nodule sizes (Reader 0), estimated measurements for four different readers were simulated, which were all rounded to the nearest millimetre. The parameters used to represent the accuracy and precision of the consensus of radiologists (Reader 1), AI alone (Reader 2), AI-assisted radiologist (Reader 3), and lone radiologist (Reader 4) are presented in Table 1, alongside other inputs used in the simulations. Further details on the simulated readers are presented in Appendix S1. Simulations were performed in RStudio 4.1.0.^30^

**Table 1.** Model inputs for the nodule size and growth simulations of the screening population.

Input name	Input value	Source/assumption
**Number of nodules simulated**	1 000 000	Assumption
**Solid nodules**		
Distribution of baseline nodule diameter	Log-normal (meanlog = −0.51, sdlog = 1.97^2^) + 3	Based on Hwang et al. (2021)^20^
Percentage that are solid	93.9%	Hwang et al. (2021)^20^
Diameter (mm)	Median = 3.6; IQR = 1.9	Hwang et al. (2021)^20^
Actual diameter after exclusion of nodules >30mm	Median = 3.3, mean = 5.5	Recalculation of data by Hwang et al. (2021)^20^
Growth curve distribution	Gompertz	Treskova (2017)^22^
% of nodules with clear features of being benign for nodules >= 5 mm	10%	Kozuka et al. 2020^23^ Veronesi et al. (2008)^24^ Rinaldi et al. (2010)^25^ Lancaster et al. (2021)^26^
% of malignant nodules at the start of the simulation	≥3 mm and <6 mm: 0.9% ≥6 mm and <8 mm: 1.1% ≥8 mm and ≤30 mm: 9.4%	Horeweg et al. (2014)^27^
% of cancerous nodules measured ≥ 8 mm referred at initial CT	91.8%	Al-Ameri et al. (2015)^4^
% of non-cancerous nodules measured ≥ 8 mm referred at initial CT	17.1%	Al-Ameri et al. (2015)^4^
**Sub-solid nodules**		
Distribution of baseline nodule diameter	Log-normal (Part-solid: meanlog = 2.19, sdlog = 0.87^2^ Non-solid: meanlog = 1.03, sdlog = 1.13^2^) + 3	Based on Hwang et al. (2021)^20^
Percentage that are sub-solid	6.1%	Hwang et al. (2021)^20^
Diameter (mm)	4:5 ratio combination of: Part-solid: Median = 11.9; IQR = 11.1 Non-solid: 5.8; IQR = 4.7	Hwang et al. (2021)^20^
Growth curve distribution	Linear	Assumption, using Kakinuma et al.^28^
% of nodules with clear features of being benign for nodules ≥ 5 mm	10%	Kozuka et al. (2020)^23^ Veronesi et al. (2008)^24^ Rinaldi et al. (2010)^25^ Lancaster et al. (2021)^26^
% of malignant nodules at the start of the simulation	< 5 mm: 0.4% ≥ 5 mm and ≤ 30 mm: 3.6%	Horeweg et al. (2014)^27^
**Accuracy of AI and Radiologists in measuring diameter for both nodule types**		
Consensus of radiologists (Reader 1)	*µ* = 0 mm SD = 0.1 mm	Assumption
AI alone (Reader 2)	Overmeasure nodules: *µ* = + 0.234 mm SD = 0.771 mm	Martins Jarnalo et al. (2021)^19^
AI-assisted radiologist (Reader 3)	Overmeasure nodules: *µ* = + 0.182 mm SD = 0.639 mm	Martins Jarnalo et al. (2021)^19^
Manual measurement (Reader 4)	Undermeasure nodules: *µ* = −0.770 mm SD = 0.959 mm^a^	Xie et al. (2013)^29^

a SD was not reported in Xie et al. (2013),^29^ assumed to be 1.5 times the SD of Reader 3.

Abbreviations: AI = artificial intelligence; IQR = interquartile range; SD = standard deviation.

### Nodule growth assumptions

We used the true nodule sizes as the baseline nodule diameter and applied growth curves to simulate how cancerous nodules grew over 24 months for surveillance of solid nodules, and 48 months for surveillance of sub-solid nodules, in line with the 2015 BTS guidelines.^4^ Additional details are provided in Appendix S1. Benign nodules did not grow.

### Nodule management following detection

Once a lung nodule is detected, its management depends primarily on its size and morphology. The nodule stratification in our simulation is a simplification of the 2015 BTS guidelines,^4^ utilizing elements that could be modelled on available data:

Solid nodules with a diameter measured under 5 mm do not require any further action and are discharged. 10% of nodules ≥ 5 mm at baseline are estimated to show clear benign features and are immediately discharged.^23–26^ Remaining nodules measuring 5-6 mm are assigned to a CT scan 1-year post-baseline. Nodules with diameter ≥ 6 mm are assigned to 3-month post-baseline CT scan. At later scans, volume doubling time (VDT) was calculated using each reader’s estimated diameter and assuming spherical form. If VDT was > 400 days at the 3-month scan, then it is assigned to 1-year CT. If estimated VDT ≤ 400 days at any scan, that person is assigned to definitive management (DM). A VDT between 400 and 600 days at year one led to a second CT scan at year two. Nodules were discharged if VDT > 600 days at 1 year, or > 400 days at 2 years. Cancerous nodules measuring ≥ 8 mm at baseline had a 91.8% chance of being referred for DM (due to Herder malignancy risk ≥ 10%), with a 17.1% chance for non-cancerous nodules of the same size.^31^

Sub-solid nodules measured under 5 mm are discharged. Again, 10% of nodules ≥ 5 mm show clearly benign features and are discharged.^23–26^ Remaining sub-solid nodules are subject to repeat CT scans at three months, and years one, two and four. Nodules are determined likely to be cancerous if an increase in diameter > 2 mm since the previous assessment is detected, otherwise are monitored until discharge at 4 years.

We assumed any nodules that are estimated to have shrunk by a reader were discharged.

## Results

### Summary of simulated nodule sizes

Our final dataset contained 1 000 000 simulated scans of risk-dominant nodules with true size at baseline between 3 and 30 mm in diameter, of which 93.9% were solid nodules.

**Figure 1.**
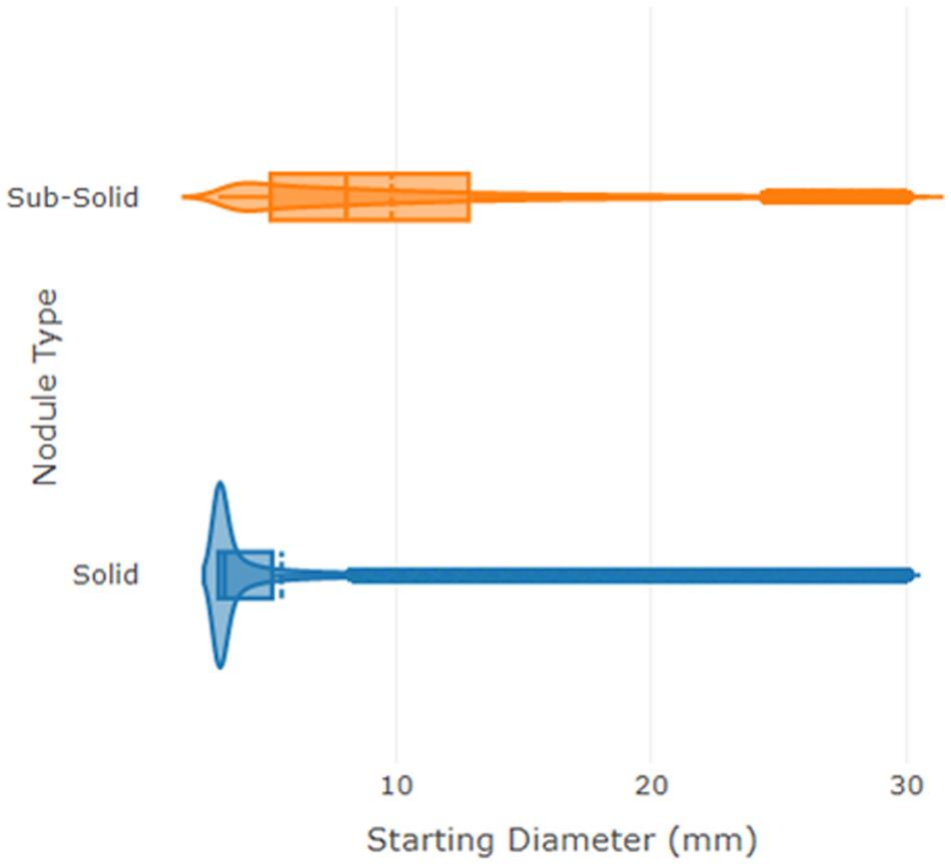
Violin plot showing the differences in distributions of simulated nodule diameters at baseline.

The median [IQR] starting diameter for solid nodules is 3.27 mm (3.03 mm, 5.13 mm), with mean 5.5 mm. For subsolid nodules, the median was 8.03 mm (5.07 mm, 12.84 mm), with mean 9.81 mm. Figure 1 is a violin plot showing the resulting log-normal distributions of the simulated solid and sub-solid nodule diameters for the screening population with corresponding boxplots showing how the medians and IQRs differ.

### Nodule growth results

**Figure 2.**
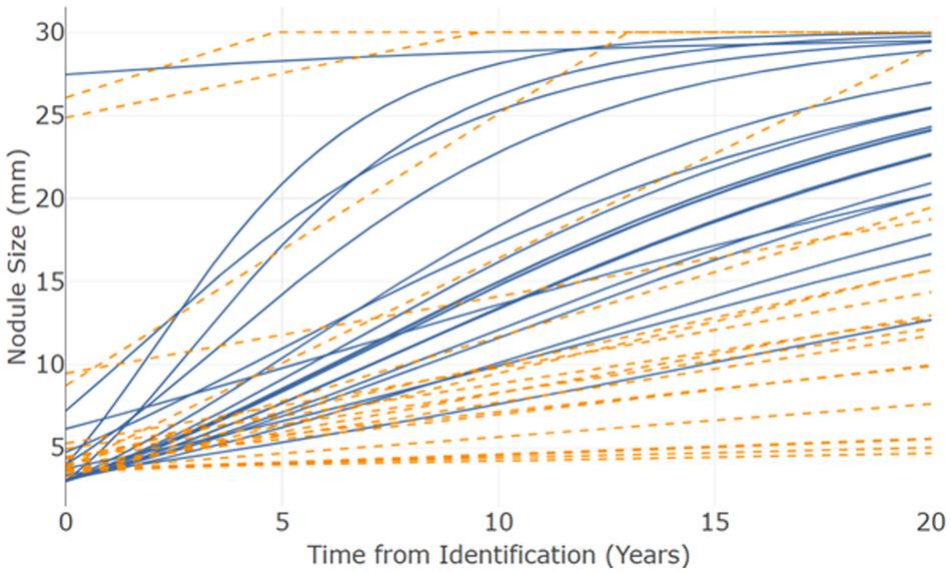
Nodule growth over time for 20 solid and 20 sub-solid cancerous nodules (solid nodules = blue; sub-solid nodules = orange-dash).

Figure 2 provides a visual representation of nodule growth in our simulation. Solid nodules followed a curved Gompertz growth function, and sub-solid nodules a linear growth function. Sub-solid growth was on average slower than solid growth.

### Assignment of nodules to different management options based on different readers

Table 2 reports the simulated number of patients assigned to DM, discharge or CT surveillance over the four-year simulation period for the different readers.

**Table 2.** The proportion of base case simulated nodules assigned to DM, discharge, or CT surveillance at each follow-up timepoint.

	Initial screening				3 months			12 months			24 months			48 months	
Mode	DM (%)	DC (%)	To 3- month CT (%)	To 12- month CT (%)	DM [cum %]	DC [cum %]	To 12- month CT	DM [cum %]	DC [cum %]	To 24- month CT	DM [cum %]	DC [cum %]	To 48- month CT	DM [cum %]	DC [cum %]
Screening population (Cancerous nodules) *N* = 22 516															
**R 0**	12 491 (55.5%)	6277 (27.9%)	2285 (10.1%)	1463 (6.5%)	346 [57.0%]	0 [27.9%]	1939	381 [58.7%]	949 [32.1%]	2072	43 [58.9%]	420 [34.0%]	1609	215 [59.9%]	1394 [40.1%]
**R 1**	12 605 (56.0%)	5977 (26.5%)	2333 (10.4%)	1601 (7.1%)	270 [57.2%]	4 [26.6%]	2059	514 [59.5%]	943 [30.8%]	2203	140 [60.1%]	414 [32.6%]	1649	165 [60.8%]	1484 [39.2%]
**R 2**	12 597 (55.9%)	5408 (24.0%)	2542 (11.3%)	1969 (8.7%)	443 [57.9%]	199 [24.9%]	1900	683 [60.9%]	1173 [30.1%]	2013	227 [62.0%]	293 [31.4%]	1493	217 [62.9%]	1276 [37.1%]
**R 3**	12 610 (56.0%)	5607 (24.9%)	2478 (11.0%)	1821 (8.1%)	382 [57.7%]	160 [25.6%]	1936	678 [60.7%]	1059 [30.3%]	2020	216 [61.7%]	262 [31.5%]	1542	179 [62.5%]	1363 [37.5%]
**R 4**	11 709 (52.0%)	6346 (28.2%)	2920 (13.0%)	1541 (6.8%)	950 [56.2%]	201 [29.1%]	1769	695 [59.3%]	950 [33.3%]	1665	226 [60.3%]	184 [34.1%]	1255	236 [61.4%]	1019 [38.6%]
Screening population (Benign nodules) *N* = 977 484															
**R 0**	19 889 (2.0%)	734 672 (75.2%)	86 412 (8.8%)	136 511 (14.0%)	0 [2.0%]	0 [75.2%]	86 412	0 [2.0%]	183 236 [93.9%]	39 687	0 [2.0%]	0 [93.9%]	39 687	0 [2.0%]	39 687 [98.0%]
**R 1**	21 751 (2.2%)	699 280 (71.5%)	97 911 (10.0%)	158 542 (16.2%)	2555 [2.5%]	3729 [71.9%]	91 627	0 [2.5%]	204 202 [92.8%]	45 967	0 [2.5%]	2902 [93.1%]	43 065	0 [2.5%]	43 065 [97.5%]
**R 2**	22 771 (2.3%)	631 441 (64.6%)	112 624 (11.5%)	210 648 (21.6%)	16 359 [4.0%]	30 319 [67.7%]	65 946	6190 [4.6%]	208 674 [89.0%]	61 730	729 [4.7%]	17 503 [90.8%]	43 498	591 [4.8%]	42 907 [95.2%]
**R 3**	22 380 (2.3%)	657 012 (67.2%)	107 522 (11.0%)	190 570 (19.5%)	14 818 [3.8%]	24 177 [69.7%]	68 527	3439 [4.2%]	195 297 [89.7%]	60 361	233 [4.2%]	15 956 [91.3%]	44 172	161 [4.2%]	44 011 [95.8%]
**R 4**	19,758 (2.0%)	728,563 (74.5%)	85,338 (8.7%)	143,825 (14.7%)	13,927 [3.4%]	21,316 [76.7%]	50,095	8,247 [4.3%]	136,927 [90.7%]	48,746	1,636 [4.5%]	12,083 [92.0%]	35,027	1,329 [4.6%]	33,698 [95.4%]

R 0 = Reader 0; True nodule diameter size; R 1 = Reader 1; Consensus of 3 radiologists; R 2 = Reader 2; stand-alone AI; R 3 = Reader 3; Radiologist assisted by AI; R 4 = Reader 4; Manual measurement.

Abbreviations: CT = Send to CT surveillance at next timepoint; cum = cumulative; DM = definitive management; DC = discharged.

At the end of the four-year period and applying the classification algorithm to the estimated nodule sizes, the different readers (R0-R4) perform similarly with ∼60% of cancerous nodules referred on for DM and 95% of benign nodules being discharged.

Differences between the readers are more clear within the first 12 months following the initial CT scan. Following the first scan, the AI-related readers (R2 and R3) discharged fewer cancerous nodules (24.0% and 24.9%, respectively) than an unassisted radiologist (R4; 28.2%) and referred more for DM (55.9% and 56.0% vs 52.0%). These absolute differences reduce over time as further scans are taken for people recommended for further CT surveillance, however, there is additional benefit for those whose disease is detected earlier.

For people with non-cancerous nodules, at baseline the AI-related readers (R2 and R3) results in fewer discharges (64.6% and 67.2%) compared to manual measurement (R4; 74.5%), and considerably more are referred for additional CT monitoring (33.1% and 30.5% vs 23.4%). Again, this difference reduces over time, however, there is obvious benefit for those rightfully discharged earlier without incurring additional CT scans and associated anxiety.

Table 3 shows the comparison of the sensitivity and specificity for each reader across the solid, sub-solid and combined populations, where the sensitivity is for the correct referral of a cancerous nodule and the specificity is for the correct discharge of a benign nodule. It also gives the mean follow-up time for people with nodules under the direction of each reader. Radiologist measurement assisted by AI-derived software increased the proportion of patients with malignant nodules being referred to DM compared to manual measurement in a population with solid and sub-solid nodules (62.5% vs 61.4%). AI-assisted radiologist also discharged slightly more people with benign solid nodules (95.8% vs 95.4%). Across all populations, manual measurement is associated with the lowest mean follow-up time, with the AI-related readers having the highest. The difference is most noticeable in the sub-solid population. Detection of sub-solid cancers appeared positively correlated with the magnitude of bias and variance associated with a reader, however, this was also associated with a lower specificity.

**Table 3.** Accuracy for detection of cancerous nodules (base case and separately for solid and sub-solid nodules) at the end of 48-month follow-up.

Analysis	Sensitivity	Specificity	Mean (SD) surveillance time (months)
**Combined solid and sub-solid nodules**			
Reader 0 (True size)	0.599	0.980	4.21 (10.21)
Reader 1 (Consensus)	0.608	0.975	4.72 (10.60)
Reader 2 (AI alone)	0.629	0.952	5.35 (10.84)
Reader 3 (Concurrent AI)	0.625	0.958	5.12 (10.86)
Reader 4 (Manual measurement)	0.614	0.954	3.95 (9.88)
**Solid nodules only** ^a^			
Reader 0 (True size)	0.635	0.978	2.37 (4.79)
Reader 1 (Consensus)	0.648	0.974	2.74 (5.12)
Reader 2 (AI alone)	0.661	0.952	3.37 (5.77)
Reader 3 (Concurrent AI)	0.661	0.956	3.10 (5.61)
Reader 4 (Manual measurement)	0.636	0.934	2.29 (5.01)
**Sub-solid nodules only**			
Reader 0 (True size)	0.168	1.000	32.65 (22.37)
Reader 1 (Consensus)	0.123	1.000	35.36 (21.13)
Reader 2 (AI alone)	0.249	0.962	35.94 (20.52)
Reader 3 (Concurrent AI)	0.195	0.988	36.31 (20.49)
Reader 4 (Manual measurement)	0.323	0.913	29.61 (22.73)

a Follow-up of solid nodules ends at 24 months.

Exploratory analyses showed that the sensitivity of all readers was affected by the initial nodule size. Alternative sources of reader accuracy information showed consistency, except for Reader 4 where relative sensitivity was superior to other readers, but specificity was inferior. Removing original reader bias and maintaining their respective deviation showed benefit of AI readers in terms of reaching the correct outcome sooner, without sacrificing long term specificity.

## Discussion

In this simulation study, we performed a comprehensive analysis of lung nodule size measurements by different readers (AI-derived software alone, radiologist assisted by AI-derived software, and unassisted radiologist) based on empirical evidence reported in the literature. We estimated the potential impact of differential nodule size measurements between the readers on subsequent management of lung nodules according to simplified 2015 BTS guidelines and on the sensitivity and specificity for lung cancer detection. This was achieved by incorporating modelling of the growth of malignant nodules into our simulation. Our results suggest that AI-assisted reading results in the detection of additional cancerous nodules but at the cost of additional monitoring of patients with non-cancerous nodules compared with manual measurement which was driven by an overestimation of nodule size, which persisted across various sensitivity analyses. The impact suggested by our simulation results is consistent in direction and magnitude with findings from two previous multiple-case multiple-reader studies. According to Park et al., AI-based software use increased the sensitivity of a whole read for the detection of lung cancer from 85.2% to 91.6% (average, +6.4%), but decreased specificity from 81.9% to 76.3% (average, -5.6%).^13^ With AI-based software use, the readers tended to upstage (average, 12.3%) rather than downstage Lung-RADS categories (average, 4.4%) compared to manual reading, with 34.2% classed as screen-positive (Lung-RADS ≥3) with compared to 28.5% without software use.^13^ Jacobs et al. found that the proportion of scans with positive screening results (Lung-RADS category ≥3) increased from 53% to 66%.^12^ The non-cancerous population is much larger and on a population level it is a tricky trade-off between better and earlier cancer detection vs additional patient stress and healthcare resource use incurred from the additional monitoring. For sub-solid nodules, concurrent AI detected fewer cancerous cases, potentially an artefact of algorithm limitations.

One potential advantage of measurement of nodule size by software is the ability to directly measure the 3D nodule volume instead of manual measurement of nodule diameter on a 2D plane. The latter provides a good estimation of nodule volume if a nodule is perfectly spherical in shape; however, use of diameter measurement to estimate nodule volume is more susceptible to measurement error if a nodule has an irregular shape, as the measured diameter varies depending on which cross-sectional plane of the CT images is used. This potential advantage of volumetry assessment is reflected in the 2015 BTS guidelines^4^: solid nodules are discharged after the 12-month CT scan if they are stable based on volumetry, but are assigned to a further 24-month CT scan if they are stable based on diameter measurement alone. However, as volumetry assessment is not a feature unique to AI-derived software (ie, there exists software which is capable of carrying out semi-automated volumetry in the process of manual assessment of the nodule, but which does not involve an AI-derived algorithm), and as our focus was to estimate the advantage specifically afforded by AI-derived software, we made a simplifying assumption that the readers would each measure nodule diameter at every scan, and have respective volumetric estimates on the assumption of spherical form.

However, accuracy of AI-assisted measurement might vary between software packages and even software versions, characteristics of lesions and CT acquisition parameters.^14^^,^^32^^,^^33^ Thus, it is important to verify the performance in one own’s clinical setting and, wherever possible, to use the same software, version, CT scanner and CT acquisition parameters for nodule surveillance. The core technology for AI-based nodule measurement is nodule segmentation. A recent systematic review^21^ found that nodule segmentation might fail or is rejected by the reader in up to 57% of nodules (8 studies) and failure rates seem to be higher in pure ground glass nodules (34%) and part-solid nodules (19.7%) compared to solid nodules (7%) (1 study). Manual modifications of the segmentation were required in 29% to 59% of nodules (2 studies). Therefore, even after implementation of AI-based size measurement, visual verification of the nodule segmentation by human readers should be recommended.

The use of AI for nodule detection and measurement raises a number of ethical considerations. Whenever there is a discrepancy between the radiologist and AI reads, the radiologist is required to dismiss or accept the AI output. In this case, two scenarios deserve special consideration. The first is when the AI detects a nodule and the radiologist dismisses it as insignificant but the nodule subsequently turns out to be malignant. The patient may then justifiably question why the AI was overridden and may feel they have been unfairly disadvantaged. The second scenario is when the AI either determines the nodule has falsely grown or is falsely stable due to measurement error. The determination of false growth may lead to overdiagnosis and unnecessary/invasive investigations such as biopsy/resection whilst the determination of false stability may lead to delayed diagnosis. In either of these instances the patient may question why an inaccurate AI read has caused potential harm. Another ethical consideration with the use of AI in lung nodule detection and measurement is the potential for unsuspected coding errors which lead to inaccuracies and systematic under or over-measurement that may only become evident after many scans have already been read, potentially causing patient harm. More sinister is the potential for AI algorithms (which often now reside on cloud-based servers) to be maliciously hacked in order to generate false results. Therefore, quality control/algorithm testing is essential (particularly after software updates) and cyber security is also paramount.

There are several strengths and limitations to the approach taken in this article. This article uses empirical data to simulate the baseline nodule diameter measurements by different readers that would be seen in practice. This enhances the clinical relevance of our simulation and sets a benchmark against which further evaluation can be compared when more empirical evidence becomes available in the future. This approach provides a valuable insight into the potential impact of integrating AI into radiological practice, highlighting the trade-off between potential benefits and drawbacks.

While the simulation itself provides valuable insights, by nature it simplifies the complexities of real-world clinical scenarios, which means it would not account for every aspect of actual cases. For example, we did not model the possibility of “vanishing nodules” where abnormalities mistaken for nodules were initially detected and would not appear on future scans. The generalizability of the inputs to UK population and care may be limited as most studies informing model parameters were conducted in other countries.

The assumptions made in the simulations may not fully capture the complexities of the disease or clinical decision making and could, therefore, impact on the generalizability of the findings to clinical settings. The benefits of AI assistance may be underestimated as AI-derived software may offer benefit independent of diametric measurement precision, for example, improved nodule detection, vessel suppression, or more accurate estimations of volume due to avoiding reliance on assumptions of spherical form made with diameter-based readings. AI assistance may also reduce the turnaround time for reporting abnormal CT scan readings by automating the identification and measurement of lung nodules,^34^ but could increase reading time by detecting more lung nodules that need to be assessed.^35^ The performance of AI-derived software may further improve over time, and, therefore, provide additional benefit to that demonstrated in this study. The relatively low prevalence of cancerous nodules highlights the need for a dual focus in future image analysis—although studies are typically focussed on identifying cancerous nodules, it is also important to identify non-cancerous cases rapidly and correctly. Getting this right will enhance the adoption of AI by healthcare systems. It is also vital to generate additional real-world evidence of relative efficacy to support studies like ours that inform health technology assessment decision making. It is possible that existing nodule management guidelines, such as the BTS guidelines, may need to be revised in the light of the improved precision of AI.

Using AI-based software for lung cancer nodule measurements may offer benefits such as greater accuracy and consistency, volumetric measurement, quicker assessment, earlier detection, and objective quantitative analysis.

Studies have shown that use of AI as a concurrent reader can decrease the time taken to read a scan when compared with a stand-alone radiologist.^36^^,^^37^ However, these studies focus largely on the use of AI for nodule detection. In comparison, there is a lack of studies looking at improvements in reading time that solely compare automated nodule size measurement vs manual measurement with electronic callipers. This is perhaps because it is self-evident that an automated measurement that is pre-embedded onto the scan images (typically as an image overlay) will be quicker than a radiologist having to manually select and place a calliper; although the relatively small marginal time gain from automating this task will clearly be magnified the more nodules that a patient has. The most important gain in efficiency is the speed with which changes in nodule size between scans, and, therefore, growth, can be assessed using AI, and the rapidity with which this can be translated into clinically relevant predictors of malignancy such as the Brock score, which is heavily influenced by nodule size.

By reducing subjectivity and integrating data, AI assists radiologists to make informed diagnoses and treatment plans. AI technology also aids in research, standardizes care across institutions, and enhances efficiency. Collaboration between radiologists and AI developers remains crucial for effective integration into clinical practice.

While the collaboration between AI and radiologists increased the number of cancerous nodules correctly identified, it also led to a decrease in the discharge of non-cancerous nodules. This raises questions about the balance between sensitivity and specificity in AI-assisted diagnosis. While AI may be more proficient at detecting potential cancerous nodules, it may also lead to increased false positives, potentially causing unnecessary stress for patients and subjecting them to prolonged surveillance.

To address these questions and refine AI-assisted lung nodule assessment, future studies should focus on collecting data that assess not only the accuracy of detection but also the timing of detection and quantifying other potential benefits. This could help determine whether AI detects cancerous nodules earlier than traditional methods, potentially improving patient outcomes. Greater information on the relevance of nodule characteristics would allow the execution of a simulation study more representative of real-world use. AI may identify new characteristics that are not yet known. Additionally, studies should investigate the psychological and emotional impact of prolonged surveillance on patients to gauge the extent of any additional stress caused by AI-enhanced diagnosis. Those implementing AI in clinical settings should carefully consider the trade-off between sensitivity and specificity and cost-effectiveness, emphasizing the importance of comprehensive training and collaboration between AI systems and healthcare professionals to optimize their performance while minimizing potential overdiagnosis and patient anxiety. Guidelines for classifying patients may need to be redesigned with the additional features and improved accuracy of AI in mind. Cut-offs generated specifically for AI have the potential to improve sensitivity and specificity.

## Conclusion

This simulation study is the first to show the extended impact of the potential improved accuracy and precision of generic AI-assisted measurement methods on patients with either cancerous or benign nodules. AI may detect cancerous nodules faster and more frequently, but that this is at the expense of longer monitoring of those with benign nodules. Careful consideration of this balance is vital to the implementation of AI and the quest to improve our healthcare systems.

## Supplementary Material

ubae010_Supplementary_Data
