# Supplementary material for: Translating potential improvement in the precision and accuracy of lung nodule measurements on computed tomography scans by software derived from artificial intelligence into impact on clinical practice—a simulation study
Source: BJR Artif Intell. 2024 Jun 6;1(1):ubae010. doi: 10.1093/bjrai/ubae010 (PMC13045710; doi:10.1093/bjrai/ubae010)
Supplement: ubae010_Supplementary_Data [file ubae010_supplementary_data.docx]

# APPENDIX

## Details on assumptions for each Reader

### Reader 0: “True” nodule size

Reader 0 was the true nodule size. Initial nodule sizes were sample from a log-normal distribution with parameters calculated to match median and interquartile range (IQR) values from Hwang et al. (10) prior to truncation of values >30mm. The median (IQR) average transverse size of solid nodules was 3.6 mm (1.9 mm). Sub-solid nodules were further separated into part-solid and non-solid nodules in the ratio 4:5. Median (IQR) size was 11.9 mm (11.1 mm) for part-solid nodules and 5.8 mm (4.7 mm) for non-solid nodules.

### Reader 1: Consensus of radiologists

Reader 1 was the consensus of multiple radiologists and is the gold standard used in practice as the reference point in most available literature. Hence we assumed minor disagreement in estimating true size from Reader 0 measurement using a standard deviation (SD) of 0.1. Nodule measurements for this and subsequent readers was rounded to the nearest millimetre as would occur in normal clinical practice.

### Reader 2: AI-derived software alone

The measured sizes for Reader 2 deviated from the true values using a mean (SD) of +0.234 (0.771) based on the comparison between nodule sizes estimated by AI-derived software and a reference standard formed by the consensus of three experienced radiologists reported by Martins Jarnalo et al. (19).

### Reader 3: Concurrent AI (Radiologist + AI-derived software)

The inputs for Reader 3, concurrent AI, were also taken from the discrepancies reported by Martins Jarnalo et al. (8). In this case, we made the assumption that the radiologist working alongside the AI-derived software manually corrected the largest discrepancy, the 4 mm over-estimation by AI-derived software. This reflected a common error in segmentation made by AI-derived software which sometimes included non-nodule structures and therefore over-estimated nodule size. The inputs of the simulation for Reader 3 were calculated to be biased with an overestimation of 0.182 mm, with a SD of 0.639 for the discrepancy between Reader 3 and 0.

### Reader 4: Unassisted Radiologist

Inputs for unassisted Radiologist reading were taken from a phantom study (19), in which unassisted radiologists measured artificial pulmonary nodules of different sizes and density. Compared with the (known) sizes of the artificial nodules, the overall underestimation in size by unassisted radiologists for nodules of any density was 9.2 ± 6.0%, and for solid nodules the underestimation was 10.1 ± 6.9%. Using this, we calculated the overall underestimation of nodule size measurements as 0.77 mm. As SD was not explicitly reported, we assumed that the unassisted radiologist will measure nodules more imprecisely than concurrent AI, modelling the SD of Reader 4 to be 1.5 times the SD of Reader 3.

## Nodule Growth Assumptions and Formulae

For the growth of malignant solid nodules over time from baseline, we used the model developed by Treskova et al. (13), who suggested a Gompertz function with a shape and scale parameters for each individual nodule sampled from the log-normal distribution. Nodules could not grow beyond 30mm.

Nodule volume was calculated using the growth function based on the baseline nodule diameter of Reader 0, so that each nodule’s true growth is reflected in the analysis, at 3, 12, and 24 months (and 48 months for sub-solid nodules). Growth for sub-solid nodules was derived from Kakinuma et al. (23) with the assumption that the observed growth would continue linearly.

We assumed that non-cancerous sub-solid nodules are assigned to definitive management (DM) at baseline only or discharged at baseline or 48 months.

Equation 2: Sphere volume calculated using nodule diameter, making the assumption that nodules are spherical

$$Sphere volume= \frac{\pi}{6}*({Diameter)}^{3}$$

Equation 3: Sphere diameter calculated by rearranging the formula for calculating sphere volume

$$Sphere diameter= \sqrt[3]{\frac{6*(sphere volume)}{\pi}}$$

Equation 4: Formula for time in days taken to double volume of nodule

$$Volume doubling {time}_{t=i}\left( {VDT}_{t=i} \right)= {time}_{i}*\frac{\log\left( 2 \right)}{log(\frac{{Sphere volume at time}_{t=i}}{{Sphere volume at time}_{t=i-1}})}$$

Equation 5: Gompertz growth function used for the growth of solid nodules

$${Volume}_{t=i}={Volume}_{max}*{\frac{{Volume}_{t=0}}{{Volume}_{max}}}^{-{time}_{i}*alpha}$$

Where ${Volume}_{max}=14137.17$ and $alpha\sim Normal distribution(-7.765, 0.5504)$.

Equation 6: Linear growth function used for the growth of sub-solid nodules

$${Diameter}_{t=i}={Diameter}_{t=0}+2*\frac{{time}_{i}}{alpha}$$

Where $alpha\sim\log\left( Normal distribution\left( 1.639, 1.033 \right) \right).$

Subgroup Results

Solid Nodules Only - 1,000,000 starting population

|  | **Initial screening** | | | | **3 months** | | | **12 months** | | | **24 months** | | | **48 months** | |  |
| --- | --- | --- | --- | --- | --- | --- | --- | --- | --- | --- | --- | --- | --- | --- | --- | --- |
| **Mode** | **DM**  **(%)** | **DC**  **(%)** | **To 3- month CT (%)** | **To 12- month CT (%)** | **DM**  **[cum %]** | **DC**  **[cum %]** | **To 12- month CT** | **DM**  **[cum %]** | **DC**  **[cum %]** | **To 24- month CT** | **DM**  **[cum %]** | **DC**  **[cum %]** | **To 48- month CT** | **DM**  **[cum %]** | **DC**  **[cum %]** |  |
| **Screening population (Cancerous nodules) N = 22,077** | | | | | | | | | | | | | | | |  |
| **R 0** | 13,286 (60.2%) | 6,620  (30.0%) | 623 (2.8%) | 1,548  (7.0%) | 360  [61.8%] | 0  [30.0%] | 263 | 370  [63.5%] | 996  [34.5%] | 445 | 0  [63.5%] | 445  [36.5%] | 0 | 0  [63.5%] | 0  [36.5%] |  |
| **R 1** | 13,387  (60.6%) | 6,308  (28.6%) | 661  (3.0%) | 1,721  (7.8%) | 284  [61.9%] | 4  [28.6%] | 373 | 521  [64.3%] | 1,010  [33.2%] | 563 | 119  [64.8%] | 444  [35.2%] | 0 | 0  [64.8%] | 0  [35.2%] |  |
| **R 2** | 13,395  (60.7%) | 5,721  (25.9%) | 877  (4.0%) | 2,084  (9.4%) | 436  [62.6%] | 210  [26.9%] | 231 | 633  [65.5%] | 1,247  [32.5%] | 435 | 127  [66.1%] | 308  [33.9%] | 0 | 0  [66.1%] | 0  [33.9%] |  |
| **R 3** | 13,404  (60.7%) | 5,915  (26.8%) | 825  (3.7%) | 1,933  (8.8%) | 383  [62.4%] | 167  [27.5%] | 275 | 659  [65.4%] | 1,123  [32.6%] | 426 | 149  [66.1%] | 277  [33.9%] | 0 | 0  [66.1%] | 0  [33.9%] |  |
| **R 4** | 12,397  [56.2%] | 6,589  [29.8%] | 1,396  (6.3%) | 1,695  (7.7%) | 929  [60.4%] | 214  [30.8%] | 253 | 625  [63.2%] | 1,041  [35.5%] | 282 | 84  [63.6%] | 198  [36.4%] | 0 | 0  [63.6%] | 0  [36.4%] |  |
| **Screening population (Benign nodules) N = 977,923** | | | | | | | | | | | | | | | |  |
| **R 0** | 21,204 (2.2%) | 761,545  (77.9%) | 49,745  (5.1%) | 145,429 (14.9%) | 0  [2.2%] | 0  [77.9%] | 49,745 | 0  [2.2%] | 49,745  [97.8%] | 0 | 0  [2.2%] | 0  [97.8%] | 0 | 0  [2.2%] | 0  [97.8%] |  |
| **R 1** | 23,100  (2.4%) | 727,461  (74.4%) | 58,425  (6.0%) | 168,937  (17.3%) | 2,743  [2.6%] | 3,961  [74.8%] | 51,721 | 0  [2.6%] | 217,587  [97.0%] | 3,071 | 0  [2.6%] | 3,071  [97.4%] | 0 | 0  [2.6%] | 0  [97.4%] |  |
| **R 2** | 24,254  (2.5%) | 657,423  (67.2%) | 71,874  (7.3%) | 224,372  (22.9%) | 16,933  [4.2%] | 32,308  [70.5%] | 22,633 | 5,997  [4.8%] | 222,213  [93.3%] | 18,795 | 134  [4.8%] | 18,661  [95.2%] | 0 | 0  [4.8%] | 0  [95.2%] |  |
| **R 3** | 23,877  (2.4%) | 684,227  (70.0%) | 66,910  (6.8%) | 202,909  (20.7%) | 15,658  [4.0%] | 25,741  [72.6%] | 25,511 | 3,471  [4.4%] | 207,976  [93.9%] | 16,973 | 41  [4.4%] | 16,932  [95.6%] | 0 | 0  [4.4%] | 0  [95.6%] |  |
| **R 4** | 21,011  (2.1%) | 754,198  (77.1%) | 49,478  (5.1%) | 153,236  (15.7%) | 13,670  [3.5%] | 22,672  [79.4%] | 13,136 | 7,348  [4.3%] | 145,854  [94.4%] | 13,170 | 278  [4.3%] | 12,892  [95.7%] | 0 | 0  [4.3%] | 0  [95.7%] |  |
| R 0 = Reader 0; True nodule diameter size; R 1 = Reader 1; Consensus of 3 radiologists; R 2 = Reader 2; stand-alone AI; R 3 = Reader 3; Radiologist assisted by AI; R 4 = Reader 4; Manual measurement.  Green background denotes correct classification (DM for cancerous nodules, DC for benign).  Red background denotes incorrect classification (DC for cancerous nodules, DM for benign).  Abbreviations: CT = Send to CT surveillance at next timepoint; cum = Cumulative; DM = Definitive management; DC = Discharged | | | | | | | | | | | | | | | | |

Sub-Solid Nodules Only - 1,000,000 starting population

|  | **Initial screening** | | | | **3 months** | | | **12 months** | | | **24 months** | | | **48 months** | |  |
| --- | --- | --- | --- | --- | --- | --- | --- | --- | --- | --- | --- | --- | --- | --- | --- | --- |
| **Mode** | **DM**  **(%)** | **DC**  **(%)** | **To 3- month CT (%)** | **To 12- month CT (%)** | **DM**  **[cum %]** | **DC**  **[cum %]** | **To 12- month CT** | **DM**  **[cum %]** | **DC**  **[cum %]** | **To 24- month CT** | **DM**  **[cum %]** | **DC**  **[cum %]** | **To 48- month CT** | **DM**  **[cum %]** | **DC**  **[cum %]** |  |
| **Screening population (Cancerous nodules) N = 28,493** | | | | | | | | | | | | | | | |  |
| **R 0** | 0  (0.0%) | 975  (3.4%) | 27,518  (96.6%) | 0  (0.0%) | 47  [0.2%] | 0  [3.4%] | 27,471 | 766  [2.9%] | 0  [3.4%] | 26,705 | 681  [5.2%] | 0  [3.4%] | 26,024 | 3,291  [16.8%] | 22,733  [83.2%] |  |
| **R 1** | 0  (0.0%) | 746  (2.6%) | 27,747  (97.4%) | 0  (0.0%) | 27  [0.1%] | 0  [2.6%] | 27,720 | 491  [1.8%] | 0  [2.6%] | 27,229 | 497  [3.6%] | 0  [2.6%] | 26,732 | 2,493  [12.3%] | 24,239  [87.7%] |  |
| **R 2** | 0  (0.0%) | 856  (3.0%) | 27,637  (97.0%) | 0  (0.0%) | 536  [1.9%] | 0  [3.0%] | 27,101 | 1,541  [7.3%] | 0  [3.0%] | 25,560 | 1,685  [13.2%] | 0  [3.0%] | 23,875 | 3,337  [24.9%] | 20,538  [75.1%] |  |
| **R 3** | 0  (0.0%) | 781  (2.7%) | 27,712  (97.3%) | 0  (0.0%) | 225  [0.8%] | 0  [2.7%] | 27,487 | 1,087  [4.6%] | 0  [2.7%] | 26,400 | 1,255  [9.0%] | 0  [2.7%] | 25,145 | 2,991  [19.5%] | 22,154  [80.5%] |  |
| **R 4** | 0  (0.0%) | 2,645  (9.3%) | 25,848  (90.7%) | 0  (0.0%) | 1,024  [3.6%] | 0  [9.3%] | 24,824 | 2,284  [11.6%] | 0  [9.3%] | 22,540 | 2,399  [20.0%] | 0  [9.3%] | 20,141 | 3,486  [32.3%] | 16,655  [67.7%] |  |
| **Screening population (Benign nodules) N = 971,507** | | | | | | | | | | | | | | | |  |
| **R 0** | 0  (0.0%) | 317,791  (32.7%) | 653,716  (67.3%) | 0  (0.0%) | 0  [0.0%] | 0  [32.7%] | 653,716 | 0  [0.0%] | 0  [32.7%] | 653,716 | 0  [0.0%] | 0  [32.7%] | 653,716 | 0  [0.0%] | 653,716  [100.0%] |  |
| **R 1** | 0  (0.0%) | 261,882  (27.0%) | 709,625  (73.0%) | 0  (0.0%) | 0  [0%] | 0  [27.0%] | 709,625 | 0  [0%] | 0  [27.0%] | 709,625 | 0  [0%] | 0  [27.0%] | 709,625 | 0  [0.0%] | 709,625  [100.0%] |  |
| **R 2** | 0  (0.0%) | 228,529  (23.5%) | 742,978  (76.5%) | 0  (0.0%) | 7,768  [0.8%] | 0  [23.5%] | 735,210 | 9,734  [1.8%] | 0  [23.5%] | 725,476 | 9,656  [2.8%] | 0  [23.5%] | 715,820 | 9,558  [3.8%] | 706,262  [96.2%] |  |
| **R 3** | 0  (0.0%) | 235,009  (24.2%) | 736,498  (75.8%) | 0  (0.0%) | 2,420  [0.2%] | 0  [24.2%] | 734,078 | 3,026  [0.6%] | 0  [24.2%] | 731,052 | 3,023  [0.9%] | 0  [24.2%] | 728,029 | 2,963  [1.2%] | 725,066  [98.8%] |  |
| **R 4** | 0  (0.0%) | 331,243  (34.1%) | 640,264  (65.9%) | 0  (0.0%) | 18,159  [1.9%] | 0  [34.1%] | 622,105 | 22,947  [4.2%] | 0  [34.1%] | 599,158 | 22,244  [6.5%] | 0  [34.1%] | 576,914 | 21,392  [8.7%] | 555,522  [91.3%] |  |
| R 0 = Reader 0; True nodule diameter size; R 1 = Reader 1; Consensus of 3 radiologists; R 2 = Reader 2; stand-alone AI; R 3 = Reader 3; Radiologist assisted by AI; R 4 = Reader 4; Manual measurement.  Green background denotes correct classification (DM for cancerous nodules, DC for benign).  Red background denotes incorrect classification (DC for cancerous nodules, DM for benign).  Abbreviations: CT = Send to CT surveillance at next timepoint; cum = Cumulative; DM = Definitive management; DC = Discharged | | | | | | | | | | | | | | | | |

Sub-Solid Nodules Only - 1,000,000 starting population (44% of nodules measured >8mm referred to DM at 3 months, assuming all non-solid nodules over this size would be referred, as per clinical opinion that these would not be subject to further surveillance).

|  | **Initial screening** | | | | **3 months** | | | **12 months** | | | **24 months** | | | **48 months** | |  |
| --- | --- | --- | --- | --- | --- | --- | --- | --- | --- | --- | --- | --- | --- | --- | --- | --- |
| **Mode** | **DM**  **(%)** | **DC**  **(%)** | **To 3- month CT (%)** | **To 12- month CT (%)** | **DM**  **[cum %]** | **DC**  **[cum %]** | **To 12- month CT** | **DM**  **[cum %]** | **DC**  **[cum %]** | **To 24- month CT** | **DM**  **[cum %]** | **DC**  **[cum %]** | **To 48- month CT** | **DM**  **[cum %]** | **DC**  **[cum %]** |  |
| **Screening population (Cancerous nodules) N = 28,341** | | | | | | | | | | | | | | | |  |
| **R 0** | 0  (0.0%) | 924  (3.3%) | 27,417  (96.7%) | 0  (0.0%) | 7,987  [28.2%] | 0  [3.3%] | 19,430 | 589  [30.3%] | 0  [3.3%] | 18,841 | 446  [31.8%] | 0  [3.3%] | 18,395 | 2,350  [40.1%] | 16,045  [59.9%] |  |
| **R 1** | 0  (0.0%) | 706  (2.5%) | 27,635  (97.5%) | 0  (0.0%) | 8,502  [30.0%] | 0  [2.5%] | 19,133 | 374  [31.3%] | 0  [2.5%] | 18,759 | 330  [32.5%] | 0  [2.5%] | 18,429 | 1,670  [38.4%] | 16,759  [61.6%] |  |
| **R 2** | 0  (0.0%) | 808  (2.9%) | 27,533  (97.1%) | 0  (0.0%) | 9,102  [32.1%] | 0  [2.9%] | 18,431 | 1,057  [35.8%] | 0  [2.9%] | 17,374 | 1,206  [40.1%] | 0  [2.9%] | 16,168 | 2,254  [48.1%] | 13,914  [51.9%] |  |
| **R 3** | 0  (0.0%) | 734  (2.6%) | 27,607  (97.4%) | 0  (0.0%) | 8,989  [31.7%] | 0  [2.6%] | 18,618 | 755  [34.4%] | 0  [2.6%] | 17,863 | 862  [37.4%] | 0  [2.6%] | 17,001 | 2,072  [44.7%] | 14,929  [55.3%] |  |
| **R 4** | 0  (0.0%) | 2,619  (9.2%) | 25,722  (90.8%) | 0  (0.0%) | 7,945  [28.0%] | 0  [9.2%] | 17,777 | 1,595  [33.7%] | 0  [9.2%] | 16,182 | 1,728  [39.8%] | 0  [9.2%] | 14,454 | 2,540  [48.7%] | 11,914  [51.3%] |  |
| **Screening population (Benign nodules) N = 971,659** | | | | | | | | | | | | | | | |  |
| **R 0** | 0  (0.0%) | 317,222  (32.6%) | 654,537  (67.4%) | 0  (0.0%) | 190,712  [19.6%] | 0  [32.6%] | 463,725 | 0  [19.6%] | 0  [32.6%] | 463,725 | 0  [19.6%] | 0  [32.6%] | 463,725 | 0  [19.6%] | 463,725  [80.4%] |  |
| **R 1** | 0  (0.0%) | 261,490  (26.9%) | 710,169  (73.1%) | 0  (0.0%) | 203,741  [21.0%] | 0  [26.9%] | 506,428 | 0  [21.0%] | 0  [26.9%] | 506,428 | 0  [21.0%] | 0  [26.9%] | 506,428 | 0  [21.0%] | 506,428  [79.0%] |  |
| **R 2** | 0  (0.0%) | 228,149  (23.5%) | 743,510  (76.5%) | 0  (0.0%) | 214,286  [22.1%] | 0  [23.5%] | 529,224 | 7,047  [22.8%] | 0  [23.5%] | 522,177 | 7,084  [23.5%] | 0  [23.5%] | 515,093 | 6,916  [24.2%] | 508,177  [75.8%] |  |
| **R 3** | 0  (0.0%) | 234,444  (24.1%) | 737,215  (75.9%) | 0  (0.0%) | 210,684  [21.7%] | 0  [24.1%] | 526,531 | 2,186  [21.9%] | 0  [24.1%] | 524,345 | 2,227  [22.1%] | 0  [24.1%] | 522,118 | 2,207  [22.4%] | 519,911  [77.6%] |  |
| **R 4** | 0  (0.0%) | 330,572  (34.0%) | 641,087  (66.0%) | 0  (0.0%) | 190,881  [19.6%] | 0  [34.0%] | 450,206 | 16,360  [21.3%] | 0  [34.0%] | 433,846 | 15,962  [23.0%] | 0  [34.0%] | 417,884 | 15,529  [24.6%] | 402,355  [75.4%] |  |
| R 0 = Reader 0; True nodule diameter size; R 1 = Reader 1; Consensus of 3 radiologists; R 2 = Reader 2; stand-alone AI; R 3 = Reader 3; Radiologist assisted by AI; R 4 = Reader 4; Manual measurement.  Green background denotes correct classification (DM for cancerous nodules, DC for benign).  Red background denotes incorrect classification (DC for cancerous nodules, DM for benign).  Abbreviations: CT = Send to CT surveillance at next timepoint; cum = Cumulative; DM = Definitive management; DC = Discharged | | | | | | | | | | | | | | | | |

Scenario Analysis Results (original Reader results also included for reference) – see descriptions below for details of changes to reader accuracy

|  | **Initial screening** | | | | | **3 months** | | | **12 months** | | | | | **24 months** | | | **48 months** | |  |
| --- | --- | --- | --- | --- | --- | --- | --- | --- | --- | --- | --- | --- | --- | --- | --- | --- | --- | --- | --- |
| **Mode** | **DM**  **(%)** | **DC**  **(%)** | **To 3- month CT (%)** | **To 12- month CT (%)** | | **DM**  **[cum %]** | **DC**  **[cum %]** | **To 12- month CT** | **DM**  **[cum %]** | | **DC**  **[cum %]** | **To 24- month CT** | | **DM**  **[cum %]** | **DC**  **[cum %]** | **To 48- month CT** | **DM**  **[cum %]** | **DC**  **[cum %]** |  |
| **Screening population (Cancerous nodules)** | | | | | | | | | | | | | | | | | | |  |
| **R 0** | 12,491  (55.5%) | 6,277  (27.9%) | 2,285 (10.1%) | 1,463 (6.5%) | | 346  [57.0%] | 0  [27.9%] | 1,939 | 381  [58.7%] | | 949  [32.1%] | 2,072 | | 43  [58.9%] | 420  [34.0%] | 1,609 | 215  [59.9%] | 1,394  [40.1%] |  |
| **R 1** | 12,605  (56.0%) | 5,977  (26.5%) | 2,333  (10.4%) | 1,601  (7.1%) | | 270  [57.2%] | 4  [26.6%] | 2,059 | 514  [59.5%] | | 943  [30.8%] | 2,203 | | 140  [60.1%] | 414  [32.6%] | 1,649 | 165  [60.8%] | 1,484  [39.2%] |  |
| **R 2** | 12,597 (55.9%) | 5,408  (24.0%) | 2,542  (11.3%) | 1,969  (8.7%) | | 443  [57.9%] | 199  [24.9%] | 1,900 | 683  [60.9%] | | 1,173  [30.1%] | 2,013 | | 227  [62.0%] | 293  [31.4%] | 1,493 | 217  [62.9%] | 1,276  [37.1%] |  |
| **R 2a** | 12,874  (57.3%) | 4,710  (21.0%) | 2,507  (11.2%) | 2,376  (10.6%) | | 323  [58.7%] | 40  [21.1%] | 2,144 | 804  [62.3%] | | 1,170  [26.3%] | 2,546 | | 336  [63.8%] | 624  [29.1%] | 1,586 | 150  [64.5%] | 1,436  [35.5%] |  |
| **R 3** | 12,610  (56.0%) | 5,607  (24.9%) | 2,478 (11.0%) | 1,821 (8.1%) | | 382  [57.7%] | 160  [25.6%] | 1,936 | 678  [60.7%] | | 1,059  [30.3%] | 2,020 | | 216  [61.7%] | 262  [31.5%] | 1,542 | 179  [62.5%] | 1,363  [37.5%] |  |
| **R 3a** | 12,581  (56.0%) | 5,786  (25.8%) | 2,368  (10.5%) | 1,732  (7.7%) | | 317  [57.4%] | 86  [26.1%] | 1,965 | 648  [60.3%] | | 996  [30.6%] | 2,053 | | 180  [61.1%] | 313  [32.0%] | 1,560 | 157  [61.8%] | 1,403  [38.2%] |  |
| **R 4** | 11,709  (52.0%) | 6,346  (28.2%) | 2,920  (13.0%) | 1,541  (6.8%) | | 950  [56.2%] | 201  [29.1%] | 1,769 | 695  [59.3%] | | 950  [33.3%] | 1,665 | | 226  [60.3%] | 184  [34.1%] | 1,255 | 236  [61.4%] | 1,019  [38.6%] |  |
| **R 4a** | 13,306 (59.2%) | 146  (0.6%) | 5,358  (23.8%) | 3,657  (16.3%) | | 889  [63.2%] | 865  [4.5%] | 3,604 | 2,259  [73.2%] | | 1,678  [12.0%] | 3,324 | | 661  [76.2%] | 1,060  [16.7%] | 1,603 | 174  [76.9%] | 1,429  [23.1%] |  |
| **Screening population (Benign nodules)** | | | | | | | | | | | | | | | | | | |  |
| **R 0** | 19,889 (2.0%) | 734,672 (75.2%) | 86,412 (8.8%) | 136,511 (14.0%) | | 0  [2.0%] | 0  [75.2%] | 86,412 | 0  [2.0%] | | 183,236  [93.9%] | 39,687 | | 0  [2.0%] | 0  [93.9%] | 39,687 | 0  [2.0%] | 39,687  [98.0%] |  |
| **R 1** | 21,751 (2.2%) | 699,280 (71.5%) | 97,911  (10.0%) | 158,542 (16.2%) | | 2,555  [2.5%] | 3,729  [71.9%] | 91,627 | 0  [2.5%] | | 204,202  [92.8%] | 45,967 | | 0  [2.5%] | 2,902  [93.1%] | 43,065 | 0  [2.5%] | 43,065  [97.5%] |  |
| **R 2** | 22,771 (2.3%) | 631,441  (64.6%) | 112,624  (11.5%) | 210,648  (21.6%) | | 16,359  [4.0%] | 30,319  [67.7%] | 65,946 | 6,190  [4.6%] | | 208,674  [89.0%] | 61,730 | | 729  [4.7%] | 17,503  [90.8%] | 43,498 | 591  [4.8%] | 42,907  [95.2%] |  |
| **R 2a** | 25,471  (2.6%) | 553,238  (56.6%) | 134,894  (13.8%) | 263,930  (27.0%) | | 9,028  [3.5%] | 15,742  [58.2%] | 110,124 | 25  [3.5%] | | 310,890  [90.0%] | 63,139 | | 1  [3.5%] | 13,131  [91.4%] | 50,007 | 1  [3.5%] | 50,006  [96.5%] |  |
| **R 3** | 22,380  (2.3%) | 657,012  (67.2%) | 107,522  (11.0%) | 190,570  (19.5%) | | 14,818  [3.8%] | 24,177  [69.7%] | 68,527 | 3,439  [4.2%] | | 195,297  [89.7%] | 60,361 | | 233  [4.2%] | 15,956  [91.3%] | 44,172 | 161  [4.2%] | 44,011  [95.8%] |  |
| **R 3a** | 22,395  (2.3%) | 674,464  (69.0%) | 104,977 (10.7%) | 175,697  (18.0) | | 11,727  [3.5%] | 17,683  [70.8%] | 75,567 | 854  [3.6%] | | 192,465  [90.5%] | 57,945 | | 8  [3.6%] | 13,360  [91.9%] | 44,577 | 4  [3.6%] | 44,573  [96.4%] |  |
| **R 4** | 19,758  (2.0%) | 728,563  (74.5%) | 85,338  (8.7%) | 143,825  (14.7%) | | 13,927  [3.4%] | 21,316  [76.7%] | 50,095 | 8,247  [4.3%] | | 136,927  [90.7%] | 48,746 | | 1,636  [4.5%] | 12,083  [92.0%] | 35,027 | 1,329  [4.6%] | 33,698  [95.4%] |  |
| **R 4a** | 34,601  (3.5%) | 43,834  (4.5%) | 459,431  (47.0%) | 439,667  (45.0%) | | 44,470  [8.1%] | 167,268  [21.6%] | 247,693 | 8,937  [9.0%] | | 484,677  [71.2%] | 193,746 | | 37  [9.0%] | 139,262  [85.4%] | 54,447 | 2  [9.0%] | 54,445  [91.0%] |  |
| R 0 = Reader 0; True nodule diameter size; R 1 = Reader 1; Consensus of 3 radiologists; R 2 = Reader 2; stand-alone AI; R 3 = Reader 3; Radiologist assisted by AI; R 4 = Reader 4; Manual measurement.  Green background denotes correct classification (DM for cancerous nodules, DC for benign).  Red background denotes incorrect classification (DC for cancerous nodules, DM for benign).  Abbreviations: CT = Send to CT surveillance at next timepoint; cum = Cumulative; DM = Definitive management; DC = Discharged | | | | | | | | | | | | | | | | | | | |
| **Summary of accuracy parameters for readers in original and alternative source analyses** | | | | | | | | | | | | |  |  |  |  |  |  |  |
| Consensus of Radiologists (Reader 1) | | | | | µ = 0mm SD = 0.1mm | | | | | Assumption | | |  |  |  |  |  |  |  |
| AI alone (Reader 2) | | | | | Overmeasure nodules: µ = + 0.234mm SD = 0.771mm | | | | | Martins Jarnalo 2021 (19) | | |  |  |  |  |  |  |  |
| AI alone (Reader 2a) | | | | | Overmeasure nodules: Solid:  µ = + 0.969mm SD = 0.249mm  Sub-solid:  µ = + 0.884mm SD = 0.411mm | | | | | Wu 2021 (32) | | |  |  |  |  |  |  |  |
| AI-assisted Radiologist (Reader 3) | | | | | Overmeasure nodules: µ = + 0.182mm SD = 0.639mm | | | | | Martins Jarnalo 2021 (19) | | |  |  |  |  |  |  |  |
| AI-assisted Radiologist (Reader 3a) | | | | | Overmeasure nodules: µ = + 0.182mm SD = 0.448mm | | | | | Martins Jarnalo 2021 (19) – assuming further errors are corrected | | |  |  |  |  |  |  |  |
| Manual measurement (Reader 4) | | | | | Undermeasure nodules: µ = – 0.770mm SD = 0.959mm^a^ | | | | | Xie 2013 (30) | | |  |  |  |  |  |  |  |
| Manual measurement (Reader 4a) | | | | | Overmeasure nodules: Solid:  µ = + 2.38mm SD = 0.50mm  Sub-solid:  µ = + 2.38mm SD = 0.41mm | | | | | Cohen (2017) | | |  |  |  |  |  |  |  |
| ^a^: SD was not reported in Xie 2013(30), assumed to be 1.5 times the SD of Reader 3  AI, artificial intelligence; IQR, interquartile range; SD, standard deviation | | | | | | | | | | | | |  |  |  |  |  |  |  |

All Nodules - 1,000,000 starting population (using SD for readers only, no bias)

|  | **Initial screening** | | | | **3 months** | | | **12 months** | | | **24 months** | | | **48 months** | |  |
| --- | --- | --- | --- | --- | --- | --- | --- | --- | --- | --- | --- | --- | --- | --- | --- | --- |
| **Mode** | **DM**  **(%)** | **DC**  **(%)** | **To 3- month CT (%)** | **To 12- month CT (%)** | **DM**  **[cum %]** | **DC**  **[cum %]** | **To 12- month CT** | **DM**  **[cum %]** | **DC**  **[cum %]** | **To 24- month CT** | **DM**  **[cum %]** | **DC**  **[cum %]** | **To 48- month CT** | **DM**  **[cum %]** | **DC**  **[cum %]** |  |
| **Screening population (Cancerous nodules) N = 22,497** | | | | | | | | | | | | | | | |  |
| **R 0** | 12,476  (55.5%) | 6,295  (28.0%) | 2,270  (10.1%) | 1,456  (6.5%) | 337  [57.0%] | 0  [28.0%] | 1,933 | 394  [58.7%] | 933  [32.1%] | 2,062 | 34  [58.9%] | 424  [34.0%] | 1,604 | 192  [59.7%] | 1,412  [40.3%] |  |
| **R 1** | 12,587  (55.9%) | 5,986  (26.6%) | 2,321  (10.3%) | 1,603  (7.1%) | 268  [57.1%] | 4  [26.6%] | 2,049 | 512  [59.4%] | 934  [30.8%] | 2,206 | 145  [60.1%] | 417  [32.6%] | 1,644 | 141  [60.7%] | 1,503  [39.3%] |  |
| **R 2** | 12,461  (55.4%) | 5,743  (25.5%) | 2,519  (11.2%) | 1,774  (7.9%) | 461  [57.4%] | 181  [26.3%] | 1,877 | 624  [60.2%] | 1,069  [31.1%] | 1,958 | 205  [61.1%] | 261  [32.2%] | 1,492 | 217  [62.1%] | 1,275  [37.9%] |  |
| **R 3** | 12,515  (55.6%) | 5,843  (26.0%) | 2,478  (11.0%) | 1,661  (7.4%) | 426  [57.5%] | 134  [26.6%] | 1,918 | 613  [60.2%] | 979  [30.9%] | 1,987 | 203  [61.2%] | 246  [32.0%] | 1,538 | 175  [61.9%] | 1,363  [38.1%] |  |
| **R 4** | 12,311  (54.7%) | 5,522  (24.5%) | 2,646  (11.8%) | 2,018  (9.0%) | 621  [57.5%] | 265  [25.7%] | 1,760 | 753  [60.8%] | 1,216  [31.1%] | 1,809 | 262  [62.0%] | 255  [32.3%] | 1,292 | 219  [63.0%] | 1,073  [37.0%] |  |
| **Screening population (Benign nodules) N = 977,503** | | | | | | | | | | | | | | | |  |
| **R 0** | 19,944  (2.0%) | 734,303  (75.1%) | 86,583  (8.9%) | 136,673  (14.0%) | 0  [2.0%] | 0  [75.1%] | 86,583 | 0  [2.0%] | 183,477  [93.9%] | 39,779 | 0  [2.0%] | 0  [93.9%] | 39,779 | 0  [2.0%] | 39,779  [98.0%] |  |
| **R 1** | 21,714  (2.2%) | 698,919  (71.5%) | 98,125  (10.0%) | 158,745  (16.2%) | 2,572  [2.5%] | 3,703  [71.9%] | 91,850 | 0  [2.5%] | 204,495  [92.8%] | 46,100 | 0  [2.5%] | 2,903  [93.1%] | 43,197 | 0  [2.5%] | 43,197  [97.5%] |  |
| **R 2** | 22,012  (2.3%) | 666,588  (68.2%) | 103,739  (10.6%) | 185,164  (18.9%) | 15,215  [3.8%] | 26,101  [70.9%] | 62,423 | 5,738  [4.4%] | 183,838  [89.7%] | 58,011 | 721  [4.5%] | 15,297  [91.2%] | 41,993 | 557  [4.5%] | 41,436  [95.5%] |  |
| **R 3** | 21,822  (2.2%) | 680,219  (69.6%) | 101,612  (10.4%) | 173,850  (17.8%) | 13,900  [3.7%] | 21,989  [71.8%] | 65,723 | 3,318  [4.0%] | 178,530  [90.1%] | 57,725 | 247  [4.0%] | 14,552  [91.6%] | 42,926 | 169  [4.0%] | 42,757  [96.0%] |  |
| **R 4** | 22,148  (2.3%) | 644,925  (66.0%) | 109,735  (11.2%) | 200,695  (20.5%) | 17,449  [4.1%] | 33,534  [69.4%] | 58,752 | 10,211  [5.1%] | 192,014  [89.1%] | 57,222 | 1,857  [5.3%] | 16,108  [90.7%] | 39,257 | 1,443  [5.4%] | 37,814  [94.6%] |  |
| R 0 = Reader 0; True nodule diameter size; R 1 = Reader 1; Consensus of 3 radiologists; R 2 = Reader 2; stand-alone AI; R 3 = Reader 3; Radiologist assisted by AI; R 4 = Reader 4; Manual measurement.  Green background denotes correct classification (DM for cancerous nodules, DC for benign).  Red background denotes incorrect classification (DC for cancerous nodules, DM for benign).  Abbreviations: CT = Send to CT surveillance at next timepoint; cum = Cumulative; DM = Definitive management; DC = Discharged | | | | | | | | | | | | | | | | |

Sensitivity Results – Larger Nodule Starting Size Distribution (+0.5mm vs base case)

|  | **Initial screening** | | | | **3 months** | | | **12 months** | | | **24 months** | | | **48 months** | |  |
| --- | --- | --- | --- | --- | --- | --- | --- | --- | --- | --- | --- | --- | --- | --- | --- | --- |
| **Mode** | **DM**  **(%)** | **DC**  **(%)** | **To 3- month CT (%)** | **To 12- month CT (%)** | **DM**  **[cum %]** | **DC**  **[cum %]** | **To 12- month CT** | **DM**  **[cum %]** | **DC**  **[cum %]** | **To 24- month CT** | **DM**  **[cum %]** | **DC**  **[cum %]** | **To 48- month CT** | **DM**  **[cum %]** | **DC**  **[cum %]** |  |
| **Screening population (Cancerous nodules) N = 23,152** | | | | | | | | | | | | | | | |  |
| **R 0** | 13,199  (57.0%) | 6,033  (26.1%) | 2,354  (10.2%) | 1,566  (6.8%) | 363  [58.6%] | 0  [26.1%] | 1,991 | 477  [60.6%] | 998  [30.4%] | 2,082 | 57  [60.9%] | 441  [32.3%] | 1,584 | 182  [61.7%] | 1,402  [38.3%] |  |
| **R 1** | 13,253  (57.2%) | 5,625  (24.3%) | 2,425  (10.5%) | 1,849  (8.0%) | 308  [58.6%] | 3  [24.3%] | 2,114 | 682  [61.5%] | 973  [28.5%] | 2,308 | 186  [62.3%] | 485  [30.6%] | 1,637 | 153  [63.0%] | 1,484  [37.0%] |  |
| **R 2** | 13,239  (57.2%) | 4,579  (19.8%) | 2,730  (11.8%) | 2,604  (11.2%) | 447  [59.1%] | 314  [21.1%] | 1,969 | 939  [63.2%] | 1,425  [27.3%] | 2,209 | 328  [64.6%] | 402  [29.0%] | 1,479 | 220  [65.5%] | 1,259  [34.5%] |  |
| **R 3** | 13,244  (57.2%) | 4,861  (21.0%) | 2,605  (11.3%) | 2,442  (10.5%) | 432  [59.1%] | 199  [21.9%] | 1,974 | 838  [62.7%] | 1,356  [27.7%] | 2,222 | 300  [64.0%] | 393  [29.4%] | 1,529 | 168  [64.7%] | 1,361  [35.3%] |  |
| **R 4** | 12,311  (53.2%) | 5,992  (25.9%) | 3,065  (13.2%) | 1,784  (7.7%) | 973  [57.4%] | 258  [27.0%] | 1,834 | 819  [60.9%] | 1,099  [31.7%] | 1,700 | 241  [62.0%] | 214  [32.7%] | 1,245 | 208  [62.9%] | 1,037  [37.1%] |  |
| **Screening population (Benign nodules) N = 976,848** | | | | | | | | | | | | | | | |  |
| **R 0** | 21,099  (2.2%) | 708,884  (72.6%) | 96,078  (9.8%) | 150,787  (15.4%) | 0  [2.2%] | 0  [72.6%] | 96,078 | 0  [2.2%] | 204,460  [93.5%] | 42,405 | 0  [2.2%] | 0  [93.5%] | 42,405 | 0  [2.2%] | 42,405  [97.8%] |  |
| **R 1** | 22,784  (2.3%) | 660,999  (67.7%) | 110,719  (11.3%) | 182,346  (18.7%) | 3,032  [2.6%] | 4,534  [68.1%] | 103,153 | 0  [2.6%] | 235,574  [92.2%] | 49,925 | 0  [2.6%] | 3,454  [92.6%] | 46,471 | 0  [2.6%] | 46,471  [97.4%] |  |
| **R 2** | 24,166  (2.5%) | 543,125  (55.6%) | 134,985  (13.8%) | 274,572  (28.1%) | 19,294  [4.4%] | 42,388  [59.9%] | 73,303 | 7,482  [5.2%] | 270,992  [87.7%] | 69,401 | 811  [5.3%] | 22,375  [90.0%] | 46,215 | 589  [5.4%] | 45,626  [94.6%] |  |
| **R 3** | 24,028  (2.5%) | 575,533  (58.9%) | 125,427  (12.8%) | 251,860  (25.8%) | 17,007  [4.2%] | 32,122  [62.2%] | 76,298 | 4,134  [4.6%] | 256,194  [88.4%] | 67,830 | 232  [4.6%] | 20,203  [90.5%] | 47,395 | 228  [4.7%] | 47,167  [95.3%] |  |
| **R 4** | 20,687  (2.1%) | 690,768  (70.7%) | 96,495  (9.9%) | 168,898  (17.3%) | 15,358  [3.7%] | 26,657  [73.4%] | 54,480 | 8,880  [4.6%] | 161,540  [90.0%] | 52,958 | 1,747  [4.8%] | 13,853  [91.4%] | 37,358 | 1,342  [4.9%] | 36,016  [95.1%] |  |
| R 0 = Reader 0; True nodule diameter size; R 1 = Reader 1; Consensus of 3 radiologists; R 2 = Reader 2; stand-alone AI; R 3 = Reader 3; Radiologist assisted by AI; R 4 = Reader 4; Manual measurement.  Green background denotes correct classification (DM for cancerous nodules, DC for benign).  Red background denotes incorrect classification (DC for cancerous nodules, DM for benign).  Abbreviations: CT = Send to CT surveillance at next timepoint; cum = Cumulative; DM = Definitive management; DC = Discharged | | | | | | | | | | | | | | | | |

Sensitivity Results – Smaller Nodule Starting Size Distribution (-0.5mm vs base case)

|  | **Initial screening** | | | | **3 months** | | | **12 months** | | | **24 months** | | | **48 months** | |  |
| --- | --- | --- | --- | --- | --- | --- | --- | --- | --- | --- | --- | --- | --- | --- | --- | --- |
| **Mode** | **DM**  **(%)** | **DC**  **(%)** | **To 3- month CT (%)** | **To 12- month CT (%)** | **DM**  **[cum %]** | **DC**  **[cum %]** | **To 12- month CT** | **DM**  **[cum %]** | **DC**  **[cum %]** | **To 24- month CT** | **DM**  **[cum %]** | **DC**  **[cum %]** | **To 48- month CT** | **DM**  **[cum %]** | **DC**  **[cum %]** |  |
| **Screening population (Cancerous nodules) N = 17,762** | | | | | | | | | | | | | | | |  |
| **R 0** | 11,963  (67.4%) | 2,407  (13.6%) | 2,025  (11.4%) | 1,367  (7.7%) | 281  [68.9%] | 0  [13.6%] | 1744 | 375  [71.0%] | 844  [18.3%] | 1,892 | 30  [71.2%] | 406  [20.6%] | 1,456 | 178  [72.2%] | 1,278  [27.8%] |  |
| **R 1** | 12,013  (67.6%) | 2,169  (12.2%) | 2,088  (11.8%) | 1,492  (8.4%) | 246  [69.0%] | 0  [12.2%] | 1,842 | 480  [71.7%] | 833  [16.9%] | 2,021 | 135  [72.5%] | 402  [19.2%] | 1,484 | 123  [73.2%] | 1,361  [26.8%] |  |
| **R 2** | 11,983  (67.5%) | 1,879  (10.6%) | 2,210  (12.4%) | 1,690  (9.5%) | 335  [69.4%] | 166  [11.5%] | 1,709 | 629  [72.9%] | 969  [17.0%] | 1,801 | 191  [74.0%] | 270  [18.5%] | 1,340 | 180  [75.0%] | 1,160  [25.0%] |  |
| **R 3** | 12,024  (67.8%) | 1,979  (11.1%) | 2,200  (12.4%) | 1,549  (8.7%) | 352  [69.7%] | 121  [11.8%] | 1,727 | 532  [72.7%] | 930  [17.1%] | 1,814 | 174  [73.7%] | 246  [18.4%] | 1,394 | 145  [74.5%] | 1,249  [25.5%] |  |
| **R 4** | 11,291  (63.6%) | 2,521  (14.2%) | 2,599  (14.6%) | 1,351  (7.6%) | 800  [68.1%] | 169  [15.1%] | 1,630 | 618  [71.6%] | 828  [19.8%] | 1,535 | 206  [72.7%] | 181  [20.8%] | 1,148 | 215  [73.9%] | 933  [26.1%] |  |
| **Screening population (Benign nodules) N = 982,238** | | | | | | | | | | | | | | | |  |
| **R 0** | 19,247  (2.0%) | 758,321  (77.2%) | 78,962  (8.0%) | 125,708 (12.8%) | 0  [2.0%] | 0  [77.2%] | 78,962 | 0  [2.0%] | 167,150  [94.2%] | 37,520 | 0  [2.0%] | 0  [94.2%] | 37,520 | 0  [2.0%] | 37,520  [98.0%] |  |
| **R 1** | 20,585  (2.1%) | 730,179  (74.3%) | 88,370  (9.0%) | 143,104  (14.6%) | 2,329  [2.3%] | 3,246  [74.6%] | 82,795 | 0  [2.3%] | 182,032  [93.3%] | 42,867 | 0  [2.3%] | 2,538  [93.6%] | 40,329 | 0  [2.3%] | 40,329  [97.7%] |  |
| **R 2** | 21,384  (2.2%) | 689,476  (70.2%) | 99,022  (10.1%) | 172,356  (17.5%) | 14,706  [3.7%] | 24,187  [72.7%] | 60,129 | 5,620  [4.2%] | 170,782  [90.0%] | 56,083 | 688  [4.3%] | 14,746  [91.5%] | 40,649 | 556  [4.4%] | 40,093  [95.6%] |  |
| **R 3** | 21,340  (2.2%) | 704,835  (71.8%) | 95,486  (9.7%) | 160,577  (16.3%) | 12,907  [3.5%] | 20,116  [73.8%] | 62,463 | 3,080  [3.8%] | 164,700  [90.6%] | 55,260 | 213  [3.8%] | 13,687  [92.0%] | 41,360 | 157  [3.8%] | 41,203  [96.2%] |  |
| **R 4** | 18,715  (1.9%) | 757,213  (77.1%) | 77,933  (7.9%) | 128,377  (13.1%) | 12,605  [3.2%] | 18,581  [79.0%] | 46,747 | 7,373  [3.9%] | 122,188  [91.4%] | 45,563 | 1,474  [4.1%] | 10,848  [92.5%] | 33,241 | 1,179  [4.2%] | 32,062  [95.8%] |  |
| R 0 = Reader 0; True nodule diameter size; R 1 = Reader 1; Consensus of 3 radiologists; R 2 = Reader 2; stand-alone AI; R 3 = Reader 3; Radiologist assisted by AI; R 4 = Reader 4; Manual measurement.  Green background denotes correct classification (DM for cancerous nodules, DC for benign).  Red background denotes incorrect classification (DC for cancerous nodules, DM for benign).  Abbreviations: CT = Send to CT surveillance at next timepoint; cum = Cumulative; DM = Definitive management; DC = Discharged | | | | | | | | | | | | | | | | |
